# A Novel Coverage Optimization Strategy Based on Grey Wolf Algorithm Optimized by Simulated Annealing for Wireless Sensor Networks

**DOI:** 10.1155/2021/6688408

**Published:** 2021-03-16

**Authors:** Yong Zhang, Li Cao, Yinggao Yue, Yong Cai, Bo Hang

**Affiliations:** ^1^School of Mathematics and Computer Science, Hubei University of Arts and Science, Xiangyang 441053, China; ^2^School of Information Engineering, Southwest University of Science and Technology, Mianyang 621010, China; ^3^Oujiang College, Wenzhou University, Wenzhou 325035, China

## Abstract

The coverage optimization problem of wireless sensor network has become one of the hot topics in the current field. Through the research on the problem of coverage optimization, the coverage of the network can be improved, the distribution redundancy of the sensor nodes can be reduced, the energy consumption can be reduced, and the network life cycle can be prolonged, thereby ensuring the stability of the entire network. In this paper, a novel grey wolf algorithm optimized by simulated annealing is proposed according to the problem that the sensor nodes have high aggregation degree and low coverage rate when they are deployed randomly. Firstly, the mathematical model of the coverage optimization of wireless sensor networks is established. Secondly, in the process of grey wolf optimization algorithm, the simulated annealing algorithm is embedded into the grey wolf after the siege behavior ends and before the grey wolf is updated to enhance the global optimization ability of the grey wolf algorithm and at the same time improve the convergence rate of the grey wolf algorithm. Simulation experiments show that the improved grey wolf algorithm optimized by simulated annealing is applied to the coverage optimization of wireless sensor networks. It has better effect than particle swarm optimization algorithm and standard grey wolf optimization algorithm, has faster optimization speed, improves the coverage of the network, reduces the energy consumption of the nodes, and prolongs the network life cycle.

## 1. Introduction

With the rapid development of wireless communication, electronic technology, computer network technology, and sensor means, wireless sensor networks (WSNs) came into being [1]. The wireless sensor network, which is composed of a large number of the sensor nodes with limited energy in a self-organizing and multihop manner cooperatively senses, collects and processes the information of the sensed objects in the coverage area of the network, and finally sends the information to the network owner [2, 3]. Therefore, it can be widely used in the modern military and urban construction, such as environmental monitoring, target tracking, battlefield monitoring, smart home, and so on [4]. However, the sensor nodes are usually randomly scattered in the air, which results in the randomness deployment of the nodes, and it is difficult to meet the monitoring of the entire area. The research of the coverage optimization of WSNs is related to a series of problems, such as the communication quality, the communication speed, and the network life cycle. Improving the coverage performance of the network can enhance the connectivity of network communication, the effectiveness of rational use of the energy, improve the quality of the network services, and promote mutual cooperation between the sensor nodes [5]. The coverage control of WSNs can reasonably allocate network resources, thereby optimizing network coverage performance. Therefore, it is of great significance to study the coverage problem of wireless sensor networks [6].

The coverage control optimization problem of wireless sensor network has become one of the hot topics in the current field. The real-time measurement of the coverage of the network plays an important role in monitoring whether there are communication and monitoring blind spots in the target area. If a blind spot is found, it can be solved by adjusting the sensor node layout or adding sensor nodes. At the same time, for more important monitoring areas, it can adopt the method of deploying more sensor nodes to improve the reliability of monitoring [7]. It can also reduce communication interference by adjusting the location of the sensor nodes. It can be seen that wireless sensor network coverage can ensure communication coverage and monitoring area coverage at the same time, which plays a vital role in the implementation of practical applications [8]. It will promote the progress of practical application work closely related to it and constantly enrich and improve the research scope of coverage control technology.

In practical applications, because of the limitations of the physical environment, the sensor nodes are mostly deployed in a random deployment mode. Therefore, there may be coverage blind areas, which cannot effectively cover the area to be monitored, thereby affecting the monitoring capability of the entire wireless network. In order to obtain more real-time monitoring data, a large number of sensor nodes need to be deployed to reduce coverage blind spots. However, due to the large scale and large density of static nodes deployed in the monitoring area, a large amount of coverage redundancy will be generated, which in turn will cause the redundancy of data collection and data transmission. Therefore, it can not only affect the reliability of the transmitted data information, but also produce some extra energy consumption output. For WSNs, the basic problem is to be able to detect whether the network coverage is optimal and make effective adjustments in a timely manner. If this problem can be effectively solved, the quality of data transmission in the network can be significantly improved, thereby reducing the waste of network resources and prolongs the network life cycle.

On the premise of ensuring the performance of network services, how can we use the least nodes to make it cover the largest area to provide the accurate data collection information and the target tracking services. The traditional method is to deploy the static nodes on a large scale to improve the coverage of the network. However, too many sensor nodes will lead to communication conflicts. Many researchers apply swarm intelligence optimization algorithm to the coverage optimization of wireless sensor networks, such as particle swarm, ant colony, artificial bee colony, and so on. However, these algorithms have the problems of low solution accuracy, slow convergence speed, and easiness to fall into local optimum when optimizing network coverage, which will lead to poor network data transmission accuracy and node redundancy. Therefore, in this paper, we propose a grey wolf algorithm optimized by simulated annealing method (SA-GWO) to effectively arrange the sensor nodes in the monitoring area to improve coverage, reduce the redundancy of the sensor node, and extend the network life cycle.

In this work, a new method of coverage optimization strategy for wireless sensor networks based on grey wolf algorithm optimized by simulated annealing method is proposed. In comparison with the current general selection approaches, the main contributions of our work in this paper can be summarized as follows:

1. Characterize the issues of a coverage control method for WSNs and formulate the problem of the coverage control algorithm.
2. Present a novel coverage optimization strategy based on grey wolf algorithm optimized by simulated annealing method.
3. Provide extensive simulation results to demonstrate the use and efficiency of the proposed coverage optimization algorithm.
4. Evaluate the performance of the proposed algorithms by comparing them with the coverage optimization algorithms of particle swarm optimization (PSO) and grey wolf optimization (GWO).

The remainder of this paper is organized as follows: In Section 2, the related works associated with the coverage optimization method for WSNs are remarked. In Section 3, the mathematical model of the coverage optimization for WSNs is introduced.  Section 4 details the working process of grey wolf algorithm optimized by simulated annealing and the application of SA-GWO algorithm in coverage optimization strategy of WSNs. In Section 5, simulation analysis and discussions are presented. Conclusions are drawn in Section 6.

## 2. Related Work

Through the study of coverage optimization, it can improve the coverage of the network, reduce the distribution redundancy of the sensor nodes, reduce the energy consumption, prolong network's lifetime, and then ensure the stability of the entire network. Some experts and scholars use the traditional algorithms to improve the coverage efficiency of WSNs. In [9], the authors proposed perimeter-based coverage optimization protocol (PeCO) for WSNs; the novelty of the approach lies essentially in the formulation of a new mathematical optimization model based on the perimeter-coverage level to schedule sensors' activities. The experiments demonstrate that the PeCO algorithm can offer longer lifetime and efficiently improve sensor network coverage. In [10], the authors proposed a localized self-deployment scheme for the deployment of randomly scattered mobile sensor nodes to cover predefined targets while maintaining connectivity with the base station in the presence of obstacles for WSNs. In [11], the authors dealt with the redundant nodes in the wireless sensor network based on grid division to improve the coverage of the network. In [12], the authors proposed a deployment of wireless sensor network nodes based on dynamic sensing distance and found the combination of sensor nodes and radius through traversal algorithm. In [13], the authors proposed a travel route planning schema with a mobile collector (TRP-MC) to find a short route that covers as many sensors as possible for WSNs; the proposed algorithm had a high coverage rate and prolonged the network's lifetime. There are many studies on the coverage optimization of these wireless sensor networks, and the overall research has also made some important breakthroughs and valuable research results.

Although the traditional algorithm has achieved good results and significant progress in the optimization of coverage, there are still some obvious deficiencies. For example, the structure of some algorithms is too complicated, resulting in the overall calculation speed being too slow to meet real-time requirements. The performance of some algorithms is too poor, resulting in a poor coverage effect, which is far from the user's service requirements. Too many parameters of certain algorithms make the network model too complicated, and the actual deployment method is often not easy to do. The swarm intelligence algorithm proposes new ideas to solve the WSNs coverage optimization problem. In recent years, a large number of scholars have applied swarm intelligence algorithms and soft computing approaches to several fields such as WSNs coverage control [14], classification algorithm [15], airport clustering, and imaging of corrosion and then studied their performance. For example, in literature [16], the authors used firefly algorithm to optimize coverage problem of WSNs; the optimization effect is better, but the algorithm as a whole is more complicated and the convergence speed is slower. A new algorithm based on particle swarm optimization (PSO) was proposed to optimize coverage problem of WSNs [17]. The algorithm had the strong global convergence ability and could quickly find the deployment model with high coverage for WSNs, but it was easy to fall into local optimization after optimization. A wireless sensor network node optimal coverage method based on improved genetic algorithm and binary ant colony algorithm was proposed in [18], the binary code expected a low intelligence of each ant, and each path corresponded to a comparatively small storage space, thus considerably improving the efficiency of computation; the proposed algorithm had a high coverage rate, thus prolonging the network lifetime efficiently. In [19], the authors proposed an artificial fish swarm algorithm WSNs coverage optimization algorithm, which had achieved better optimization results in the coverage optimization of WSNs and improved the coverage of the network, but the node coverage redundancy is higher. In [20], the authors proposed an enhanced deployment algorithm based on artificial bee colony (ABC). The ABC-based deployment is guaranteed to extend the lifetime by optimizing the network parameters and constraining the total number of deployed relays. In [21], the authors proposed a fuzzy-based procedure to cluster airports by using a fuzzy geometric point of view according to the concept of unit-hypercube. A regularization approach based on evolutionary computation was proposed to obtain an imagery reconstructing the corrosion profile [22]. In [23], the authors proposed a wireless sensor network coverage optimization model based on improved whale algorithm. The mathematic model of node coverage in wireless sensor networks was developed, and the idea of reverse learning was introduced into the original whale swarm optimization algorithm to optimize the initial distribution of the population. The proposed algorithm could effectively improve the coverage of nodes in wireless sensor networks and optimize the network performance.

However, these algorithms have some shortcomings in the coverage optimization of WSNs. For example, the poor optimization value of particle swarm optimization results in low network coverage. The ant colony algorithm involves too many parameters, forming a complex network model, which in turn causes practical deployment difficulties. In response to these problems, in this paper, we use the improved grey wolf optimization algorithm in the previous chapter to optimize the coverage of WSNs, which can effectively solve the problem of the coverage optimization of the sensor nodes for WSNs.

On the basis of summarizing the previous studies, in this paper, we analyze the mathematic model of node coverage in wireless sensor networks and propose a novel coverage optimization strategy based on grey wolf algorithm optimized by simulated annealing method.

## 3. Mathematical Model

In this paper, the probability-aware model is used to calculate the coverage rate of the network. The coverage of each sensor node in the wireless sensor network takes itself as the sensing center and has a circular area with a fixed communication radius [24, 25]. Therefore, it is difficult for all sensor nodes to solve the total coverage of the monitoring area by formula. In order to simplify the coverage problem in wireless sensor network, the area to be monitored can be discretized into *m* × *n* pixels. Assuming that there are *x* pixels covered by wireless sensor network, the coverage can be expressed as *x*/(*m* × *n*).

Suppose that the measurement radius *r* of each sensor node in WSNs is the same as the communication radius *r*_*s*_, and the coverage area of each sensor node is a circular area with the radius *r*. In this paper, we assume that the measured area of the sensor network is a two-dimensional plane *M*, which is discretized into *m* × *n* pixels. There are *N* sensor nodes in the wireless sensor network. The set of sensor nodes in the measured area is *G* = {*g*_1_, *g*_2_,…, *g*_*N*_}; the position of the *i*th sensor node *g*_*i*_ is (*x*_*i*_, *y*_*i*_). Assuming that the coordinate of the pixel *H* is (*x*_*H*_, *y*_*H*_), then the distance between the pixel and the sensor node *g*_*i*_ is

(1) $$\begin{array}{c} d\left(g_{i},H\right)=\sqrt{{\left(x_{H}-x_{i}\right)}^{2}+{\left(y_{H}-y_{i}\right)}^{2}}. \end{array}$$

Using a two-dimensional perception model, the probability of the sensor node *g*_*i*_ sensing pixel *H* is

(2) $$\begin{array}{c} p\left(g_{i},H\right)=\left\{\begin{array}{cc} 1, & d\left(g_{i},H\right)\leq r,\\ 0, & d\left(g_{i},H\right)>r. \end{array}\right. \end{array}$$

Assuming that any one sensor node can be sensed by multiple sensor nodes at the same time, the joint probability [23] that the sensor node at pixel *H* is sensed by the sensor node set *G* of wireless sensor network is

(3) $$\begin{array}{c} p\left(G,H\right)=1-\prod_{g_{i}\in G}\left[1-p\left(g_{i},H\right)\right]. \end{array}$$

The coverage rate of all the sensor nodes to be detected is defined as the ratio of the area of pixels covered by all nodes to the total monitored area [23]; that is,

(4) $$\begin{array}{c} \text{COV}\left(G\right)=\frac{\sum_{H\in m\times n}p\left(G,H\right)}{m\times n}. \end{array}$$

The optimization objective of the coverage model of WSN *s* is the maximum value of the coverage function in equation (4).

## 4. Application of SA-GWO Algorithm in the Coverage Optimization of WSNs

### 4.1. Grey Wolf Algorithm Optimized by Simulated Annealing

In this part, we first detail the basic principles, mathematical models, and the flowchart of the grey wolf optimization algorithm

After that, it is applied to the simulated annealing to optimize the grey wolf optimization algorithm.

#### 4.1.1. The Grey Wolf Optimization Algorithm

In recent years, a large number of scholars have paid more attention to grey wolf optimization algorithm (GWO). The grey wolf optimization algorithm is a new swarm intelligence algorithm proposed by Mirjalili et al. in 2014 [26, 27]. The grey wolf algorithm is inspired by the hunting behavior of wolves. It is an intelligent optimization algorithm that imitates the hierarchical system and hunting strategy in wolves. The grey wolf algorithm simulates the social class system of grey wolf and its predatory behavior and then uses the grey wolf's search, siege, and the hunting behaviors in the predation process to achieve the purpose of optimization [28]. The algorithm refers to the relationship between the natural grey wolf hunting division and the food distribution. It takes artificial wolf as the main body and adopts a collaborative path search structure based on responsibility division to abstract the engineering optimization solution process into the grey wolf hunting process. The algorithm has the advantages of simple model, fewer parameter settings, and better optimization performance. Therefore, the GWO algorithm is widely used in the multisensor training, the surface wave parameter optimization, cluster optimization, and other fields [29].

Assuming that each individual in the population is a solution, the number of grey wolves is *N*, and the search area is *D*-dimensional, where the position of the *i*th grey wolf can be expressed as *X*_*i*_=(*X*_*i*_^1^, *X*_*i*_^2^,…, *X*_*i*_^*d*^), *i* = 1, 2, ..., *N*; the parameter *N* is the population size. The individual with the largest fitness value (optimal solution) in the population is denoted as *α* (alpha), and the corresponding individuals with the fitness value ranking second (second best solution) and third (third optimal solution) are recorded as *β* (beta) and *δ* (delta). The other remaining individuals are recorded as *ω* (omega). The position of the prey corresponds is corresponding to the position of *α* wolf in the algorithm [30].

Suppose that the individual grey wolf in the population is *X*, according to the positions *X*_*α*_, *X*_*β*_, and *X*_*δ*_ of the parameters *α*, *β*, and *δ*, update their positions as follows:

(5) $$\begin{array}{c} X_{i,\alpha }^{d}\left(t+1\right)=X_{\alpha }^{d}\left(t\right)-A_{i,1}^{d}\left|C_{i,1}^{d}X_{\alpha }^{d}\left(t\right)-X_{i}^{d}\left(t\right)\right|, \end{array}$$

(6) $$\begin{array}{c} X_{i,\beta }^{d}\left(t+1\right)=X_{\beta }^{d}\left(t\right)-A_{i,2}^{d}\left|C_{i,2}^{d}X_{\beta }^{d}\left(t\right)-X_{i}^{d}\left(t\right)\right|, \end{array}$$

(7) $$\begin{array}{c} X_{i,\delta }^{d}\left(t+1\right)=X_{\delta }^{d}\left(t\right)-A_{i,3}^{d}\left|C_{i,3}^{d}X_{\delta }^{d}\left(t\right)-X_{i}^{d}\left(t\right)\right|, \end{array}$$

wherein the parameter *t* is the current iteration number and the parameters *X*_*α*_, *X*_*β*_, and *X*_*δ*_ are the prey positions. *A*_*i*_^*d*^|*C*_*i*_^*d*^*X*_*α*_^*d*^(*t*) − *X*_*i*_^*d*^(*t*)| is the enclosing step size; the calculation formula of the convergence factor *A*_*i*_^*d*^ and the swing factor *C*_*i*_^*d*^ is

(8) $$\begin{array}{c} A_{i}^{d}=2a\times {\text{rand}}_{1}-a, \end{array}$$

(9) $$\begin{array}{c} C_{i}^{d}=2{\text{rand}}_{2}. \end{array}$$

The parameters rand_1_ and rand_2_ are the random number, between [0, 1]. The parameter *a* is the control parameter; it decreases linearly from 2 to 0 with the increase of the number of iterations.

(10) $$\begin{array}{c} a=2-\frac{2t}{t_{\max}}. \end{array}$$

The parameter *t* is the current number of iterations, and the parameter *t*_max_ is the maximum number of iterations.

(11) $$\begin{array}{c} X_{i}^{d}\left(t+1\right)=\sum_{j=\alpha ,\beta ,\delta }w_{j}X_{i,j}^{d}\left(t+1\right), \end{array}$$

wherein the parameter *w*_*j*_(*j*=*α*, *β*, *δ*) represents the weight coefficients of *α*, *β*, and *δ*. The calculation formula is

(12) $$\begin{array}{c} w_{j}=\frac{f\left(X_{j}\left(t\right)\right)}{f\left(X_{\alpha }\left(t\right)\right)+f\left(X_{\beta }\left(t\right)\right)+f\left(X_{\delta }\left(t\right)\right)}. \end{array}$$

The parameter *f* (*X*_*j*_(*t*)) represents the fitness value of the *j*th individual in the *t*th generation.

#### 4.1.2. Grey Wolf Algorithm Optimized by Simulated Annealing

Although the GWO algorithm has received extensive attention, it also has some shortcomings, such as the slow convergence speed and the weak global search ability, etc., and in continuous iterations, the GWO algorithm is prone to falling into local optimality. In the process of optimizing the fitness function, the grey wolf swarm algorithm always approaches the maximum value. If the fitness function has a local extreme value, once it falls into the local optimum in the process of optimization, it cannot escape. The idea of simulated annealing can improve or eliminate this problem [31, 32]. Therefore, a grey wolf optimization algorithm optimized by simulated annealing (SA-GWO) is proposed in this paper. In the proposed algorithm, the SA-GWO algorithm is embedded in the end of the siege behavior of wolves and before the update of wolves to enhance the global optimization ability of the basic grey wolf algorithm and improve the convergence speed of the grey wolf algorithm.

The characteristic of the simulated annealing algorithm is that it can not only accept the good solutions, but also receive the bad solutions with a certain probability. In this way, when the algorithm falls into the local optimal solution, it may jump out of the local extremum. After the end of the siege of the grey wolf algorithm and before the update of the wolf pack, the simulated artificial operation can be performed on the best artificial wolf individual. The unoperated one is regarded as the original solution, and the discard of the new solution is determined by the Metropolis criterion of the simulated annealing algorithm.

(1) Perform a random perturbation within a certain range of the current position of the *i*th wolf in the population to generate a position vector *Y* = (*y*_1_, *y*_2_, ..., *y*_*n*_) in the current search space.
(2) Calculation: The fitness value of *Y* is recorded as *h*_*k*+1_, and the fitness value of the current position is recorded as *h*_*k*_. If *h*_*k*+1_ ≥ *h*_*k*_, the location is updated to *Y*. If *h*_*k*+1_ < *h*_*k*_, according to the Metropolis criterion, the state transition probability *ξ* determines whether it needs to be updated.
(i) When *h*_*k*+1_ < *h*_*k*_,
  (13) $$\begin{array}{c} \xi \left(T_{k+1}\right)=\min\left[1,\;\exp\left(-\frac{h_{k+1}-h_{k}}{h_{k+1}}\right)\right]>\text{random}\left(0,1\right), \end{array}$$
When *h*_*k*+1_ ≥ *h*_*k*_,
  (14) $$\begin{array}{c} \xi \left(T_{k+1}\right)=1. \end{array}$$
In equation (13), *ξ*(*T*_*k+*1_) is the receiving probability when the temperature is *T*_*k+*1_.
(3) Judge whether it is completed according to the constraints. If it is completed, it will enter the next step. Otherwise, start from the first step.
(4) If the cooling state has not been reached, then proceed to the first step after the cooling treatment according to the following equation:
  (15) $$\begin{array}{c} T_{i+1}=T_{i}\times \mu . \end{array}$$
In equation (15), the parameter *μ*is the temperature cooling coefficient. If the cooling state is reached, the algorithm ends.

**Figure 1:**
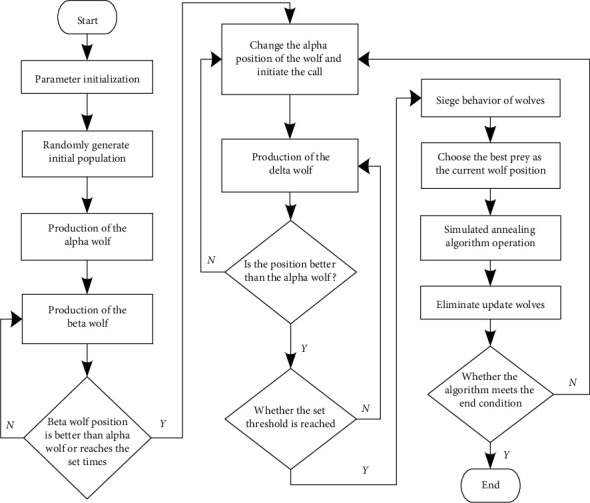
The flowchart of SA-GWO algorithm.

The flowchart of SA-GWO algorithm is shown in Figure 1.

### 4.2. Application of SA-GWO Algorithm in the Coverage Optimization of WSNs

In order to solve the coverage problem of wireless sensor networks, in this paper, we use a probabilistic perception model to calculate the coverage rate of the network. The improved grey wolf optimization algorithm is used to optimize the coverage deployment of WSNs. In this paper, the optimization goal of the SA-GWO algorithm is designed to solve the maximum value of the objective function of coverage optimization of wireless sensor networks, output the coverage after the SA-GWO optimization, and get the distribution location of all the sensor nodes in the area to be tested after the optimal deployment.

In the coverage optimization of the improved grey wolf algorithm, it is assumed that there are *M* grey wolves; each grey wolf represents a node deployment scheme that contains *N* nodes, and the location of the grey wolf is represented by *X* : *X*_*i*_ = (*x*_*i*1_, *y*_*i*1_, *x*_*i*2_, *y*_*i*2_, ..., *x*_*iN*_, *y*_*iN*_), wherein the parameter (*x*, *y*) is used to indicate the position coordinates of each sensor. Different grey wolves have different position information, and the area coverage corresponding to the position layouts is different. The basic content of the coverage optimization of the improved grey wolf algorithm is based on the grey wolf algorithm, taking the position information of the sensor node as the input value, and the coverage of the wireless sensor networks as the fitness function, which is shown in formula (4). The individual with the highest fitness value is recorded as *α*, the corresponding individuals ranked in the second and third fitness values are recorded as *β* and *δ*, and the remaining individuals are recorded as *ω*, the position of the prey corresponds to the position of the *α* wolf in the algorithm. The grey wolf population contains multiple grey wolf individuals, and each grey wolf individual has the same dimensionality. The area to be measured is a two-dimensional plane, and then the grey wolf individual's dimension is twice the number of sensor nodes. The 2*d*−1 dimension represents the abscissa of the *d*th sensor node, and the 2*d* dimension represents the ordinate of the *d*th sensor node.

The implementation steps of the coverage optimization algorithm of WSNs are as follows:

1. Set the algorithm parameters: the population size *N*, the dimension *d*, the maximum iteration number *t*_max_ and the control parameter *a*, the convergence factor *A*, the swing factor *C*, and other parameters.
2. Initialize the population *X*(*x*_1_, *x*_2_, ..., *x*_*D*_); that is, randomly generate the positions of *N* intelligent individuals, and then use the fitness function (4) to calculate the fitness value of each grey wolf in the entire population.
3. Calculate the fitness value of each grey wolf individual in the initial population, select the top three individuals of fitness value, and set them as *X*_*α*_, *X*_*β*_, and *X*_*δ*_, respectively.
4. Update the position of the grey wolf through equations (13) and (14), recompare the adaptive value, calculate the value of the control parameter *α*, and update the convergence factor *A* and the swing factor *C* according to equations (8) and (9).
5. Calculate the fitness value of each grey wolf again, and update *X*_*α*_, *X*_*β*_, *X*_*δ*_, then select the grey wolf individuals with the better fitness value.
6. Determine whether the end condition is satisfied. If it is not satisfied, the number of iterations is increased by 1, and then return to step (3). If it is satisfied, it ends and outputs *X*_*α*_.

**Figure 2:**
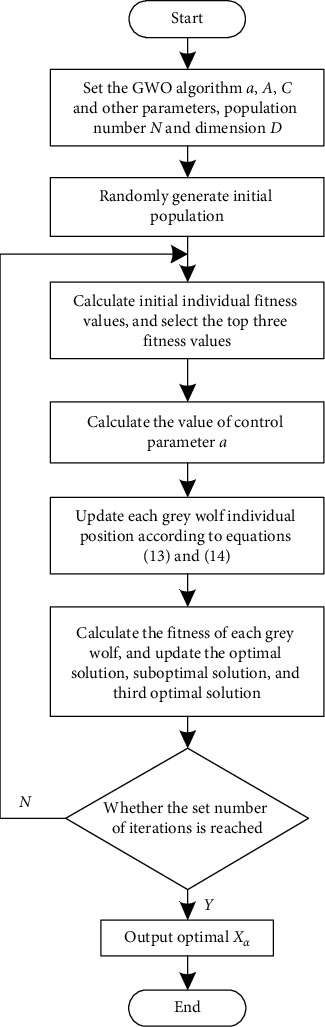
Optimal coverage optimization process based on SA-GWO algorithm.

The coverage optimization process of wireless sensor network based on SA-GWO algorithm is shown in Figure 2.

## 5. Comparison and Analysis of Algorithm Simulation

### 5.1. Simulation Environment Settings

To demonstrate the usefulness of our method, we compared with SA-GWO algorithm, GWO algorithm, and PSO algorithm by experimental simulation. We analyze the performance of the proposed algorithm by comparing the coverage effect, network coverage, network energy consumption, network life cycle, and effect of communication radius on coverage. The consistent simulation parameters are set to ensure the fairness and accuracy of the experiment result. In the comparison of algorithm coverage, each algorithm is run 50 times to obtain an average value to reflect its coverage performance. It is set that 50 sensor nodes are randomly deployed in the 100 × 100 m^2^ monitoring area, and the initial energy of each node is 1 J. All sensor network nodes are isomorphic, with the same radius of perception and the same initial energy. In order to compare and analyze the performance of the algorithm, consistent simulation conditions are used to randomly generate initial locations of sensor nodes in the monitored area. The operating environment of the experiment is Intel® Core™ i5-4210U CPU, 2.39 GHz, 8 GB memory, Windows 10, 64-bit operating system, and the simulation software is MATLAB R2016a. The experimental data in this paper is the average value of 100 times of the network simulation.

The parameters of the three algorithms are set as follows: The initial population size is 30 and the maximum number of iterations *T*_max_ is 1000. PSO algorithm sets the learning factor *c*_1_ = 2, *c*_2_ = 2 and the inertia weight factor *ω*_1_ = 0.9, *ω*_2_ = 0.4; SA-GWO algorithm sets the control parameters *α*_1_ = 0.9, *α*_2_ = 0.4, the maximum number of iterations of algorithm *T*_max_ = 1000, the initial temperature of simulated annealing algorithm *T* = 1000, and the annealing speed *θ* = 0.98.

### 5.2. Simulation Comparison and Analysis

#### 5.2.1. Comparison of Algorithm Coverage Effects

**Figure 3:**
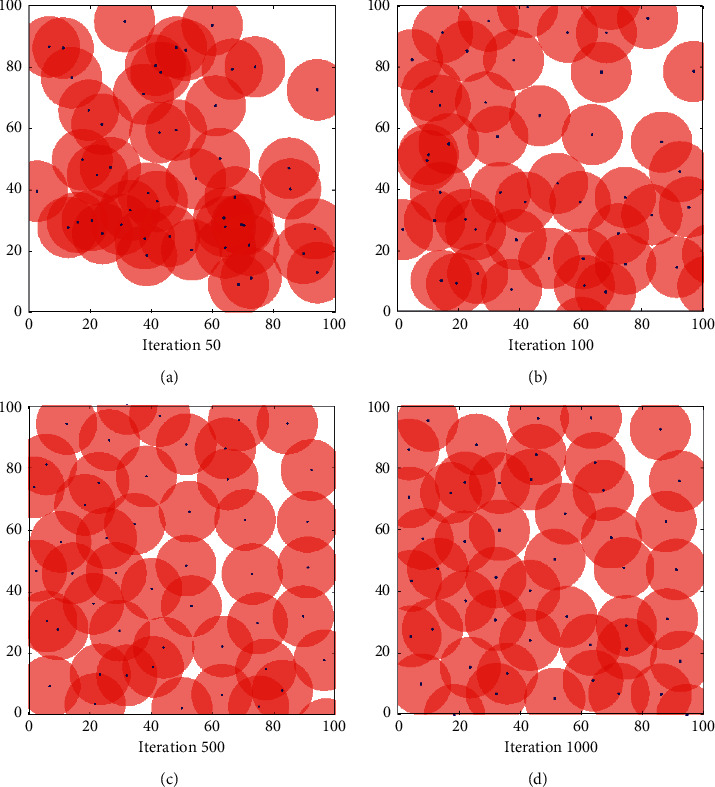
Coverage effect of PSO. (a) 50. (b) 100. (c) 500. (d) 1000.

**Figure 4:**
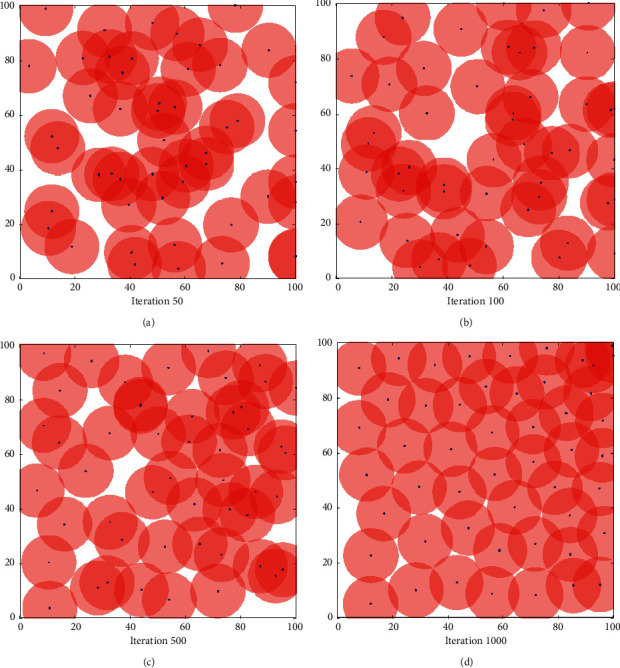
Coverage effect of GWO. (a) 50. (b) 100. (c) 500. (d) 1000.

**Figure 5:**
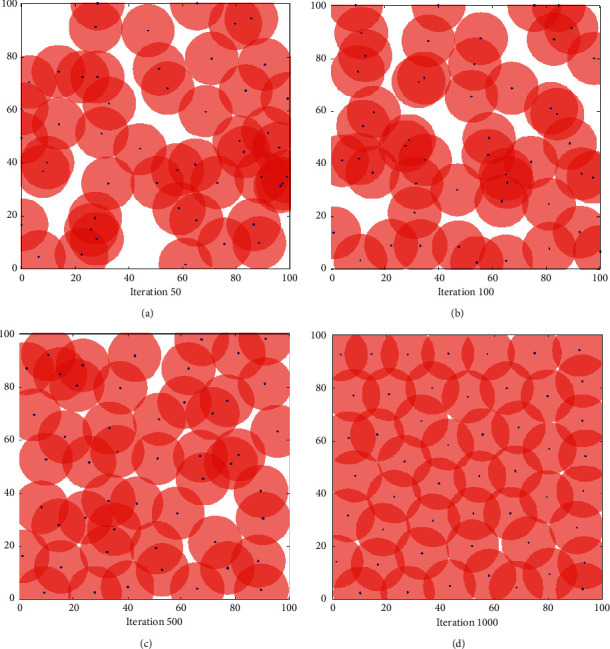
Coverage effect of SA-GWO. (a) 50. (b) 100. (c) 500. (d) 1000.

In order to verify the coverage effect of the proposed algorithm, we assume that the wireless sensor network is an isomorphic network. The simulation results are shown in Figures 3–5.  Figure 3 shows the node coverage snapshots based on particle swarm optimization (PSO), Figure 4 shows the node coverage snapshots based on grey wolf optimization algorithm (GWO), and Figure 5 shows the node coverage snapshots based on grey wolf algorithm optimized by simulated annealing (SA-GWO). In these simulation snapshots, a rectangle represents the area to be detected by the wireless sensor network, a “•” symbol represents the location of a single sensor node, and a circle represents the range that a single sensor node can cover in the monitoring area.

It can be seen from Figures 3–5 that the coverage holes of the three algorithms are gradually decreasing as the number of iterations increases. Compared with the PSO algorithm and the GWO algorithm, the SA-GWO algorithm proposed in this paper performs a relatively more uniform node distribution after optimization, which can cover more areas and increase the reliability of network transmission. In the process of algorithm simulation, under the premise of having the same number of iterations as GWO algorithm, the network coverage of the SA-GWO algorithm proposed in this paper is greatly improved. In addition, Figures 3–5 show the different node coverage results of the three algorithms for comparison after 50 iterations, 100 iterations, 500 iterations, and 1000 iterations. From a horizontal comparison point of view, at the same number of iterations, the proposed algorithm covers more grid points than other algorithms; that is, the coverage of the proposed algorithm is better than that of the other algorithms. It shows that the proposed algorithm can make the sensor nodes more evenly distributed in the detection area, thereby reducing the redundancy of the sensor nodes. From a vertical comparison point of view, with the increase of the number of iterations, the distribution of the sensor nodes is more uniform, and the coverage of the proposed algorithm is larger than the other algorithms. As can be seen from the coverage results of Figure 5, after 1000 iterations of the SA-GWO algorithm, sensor nodes can basically cover most areas, and the coverage of the sensor network is close to 100%.

#### 5.2.2. Comparison of Network Coverage

**Figure 6:**
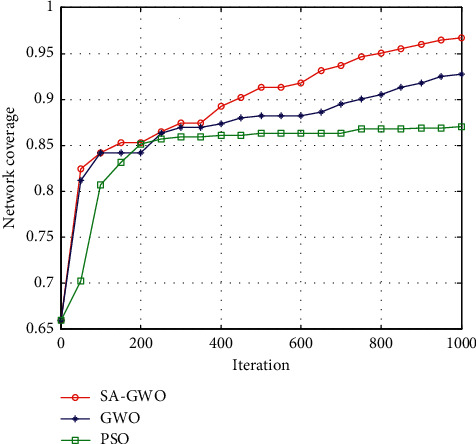
Coverage comparison of three algorithms.

The coverage of the network is one of the important measurement indicators of the quality of wireless sensor networks. The network coverage rate change curve of three network coverage algorithms under 1000 iterations is shown in Figure 6.

As can be seen from Figure 6, the three algorithms are gradually improving the network coverage as the number of iterations increases. The PSO algorithm tends to converge after 260 iterations, and the coverage rate is 86.8%. The GWO algorithm tends to converge after 600 iterations, and the coverage rate is 90.7%. For the SA-GWO algorithm proposed in this paper, because the simulated annealing algorithm is used to enhance the global optimization ability of GWO algorithm, the improved GWO algorithm can reach the convergence position faster and greatly reduce the repeated coverage. After 400 iterations, the coverage rate is 90%, and after 800 iterations of fine search, the coverage rate is 95.9%. The simulation data shows that the algorithm proposed in this paper has the advantages of larger area coverage and the better coverage effect than the PSO algorithm and the GWO algorithm under the conditions of the same population and the same number of iterations.

#### 5.2.3. Comparison of Energy Consumption

The problem of energy consumption of the network has always been a hot issue in WSNs research, which also restricts the research progress of the coverage of the network. The common methods are reasonable deployment of the sensor nodes, sleep/wake-up control, and power control in the network to maximize the energy utilization of the network and realize the optimal coverage of the network.

The energy consumption model of WSNs uses the same wireless communication model as the LEACH algorithm, and the formula for energy consumption of sensor nodes sending data is shown in the following equation:

(16) $$\begin{array}{c} E_{\text{Tx}}\left(l,d\right)=\left\{\begin{array}{cc} l\times E_{\text{elec}}+l\times \varepsilon_{fs}d^{2}, & d<d_{0},\\ l\times E_{\text{elec}}+l\times \varepsilon_{\text{amp}}d^{4}, & d\geq d_{0}. \end{array}\right. \end{array}$$

In the formula, the parameter *E*_Tx_ (*l*) is the transmission energy consumption of the length *l* of the data packet transmitted by the transmitting circuit, and the parameter *E*_Tx_(*l*, *d*) is the energy consumption of the amplifier sending the length *l* of the data packet to the distance *d*. After receiving data, the network energy consumption is as follows:

(17) $$\begin{array}{c} E_{\text{Rx}}\left(l\right)=l\times E_{\text{elec}}. \end{array}$$

The parameter *E*_Rx_(*l*) is the received energy consumption of the length *l* of the data packet transmitted by the transmitting circuit; the energy consumed by the cluster head node for data fusion *E*_AX_ is

(18) $$\begin{array}{c} E_{Ax}=l\times E_{DA}\left(\frac{1+N}{k-1}\right)=l\times E_{DA}\times \frac{N}{k}. \end{array}$$

The threshold value *d*_*0*_ is determined by the following equation:

(19) $$\begin{array}{c} d_{0}=\sqrt{\frac{\varepsilon_{\text{fs}}}{\varepsilon_{\text{amp}}},} \end{array}$$

wherein *E*_elec_ is the energy consumption of a node sending or receiving data unit, *E*_fs_ is the energy consumption parameter of the amplifier in the free space data transmission model, *E*_elec_ is the energy consumption parameter of the amplifier in the multipath attenuation data transmission model, and*ε*_amp_ is the amplifier energy consumption parameter of the multipath attenuation data transmission model. The parameter *l* is the length of the transmitted data packet, and the parameter *d*_*0*_ is the average distance between the transmitting end and the receiving end in WSNs.

**Figure 7:**
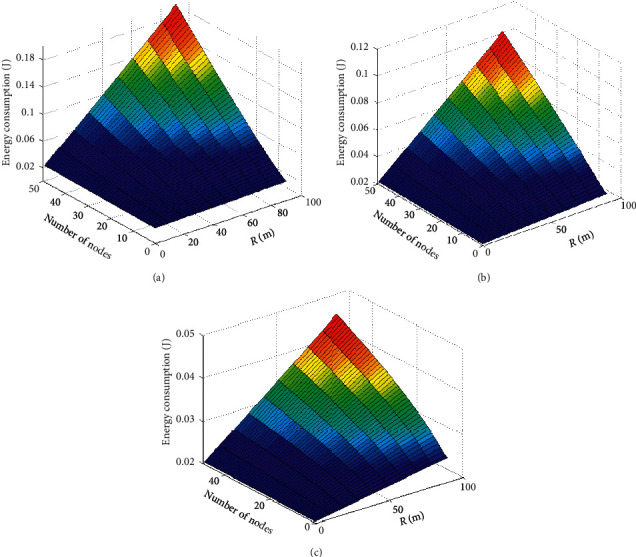
Comparison of network energy consumption of three algorithms. (a) PSO. (b) GWO. (c) SA-GWO.

The three-dimensional energy consumption comparison of the three algorithms is shown in Figure 7.

It can be seen from Figure 7 that, under the same number of sensor nodes and the same communication distance, the network energy consumption of PSO algorithm is the largest, and most of the nodes have high energy consumption, which is unbalanced, reaching 0.2 J. The network energy consumption of the GWO algorithm is relatively small, the energy consumption of most nodes is relatively high, and the energy consumption is relatively balanced. The maximum energy consumption of network nodes reaches 0.11 J. The SA-GWO algorithm proposed in this paper has the smallest network energy consumption, and the energy consumption of the sensor nodes is relatively balanced. The maximum energy consumption of the network nodes is 0.05 J. In summary, the energy consumption of PSO algorithm is the largest, and the energy consumption of GWO algorithm is larger. The energy consumption of the algorithm proposed in this paper is the smallest, which is 75% and 54.5% lower than the PSO algorithm and the GWO algorithm, respectively.

#### 5.2.4. Comparison of Network Life Cycle

**Figure 8:**
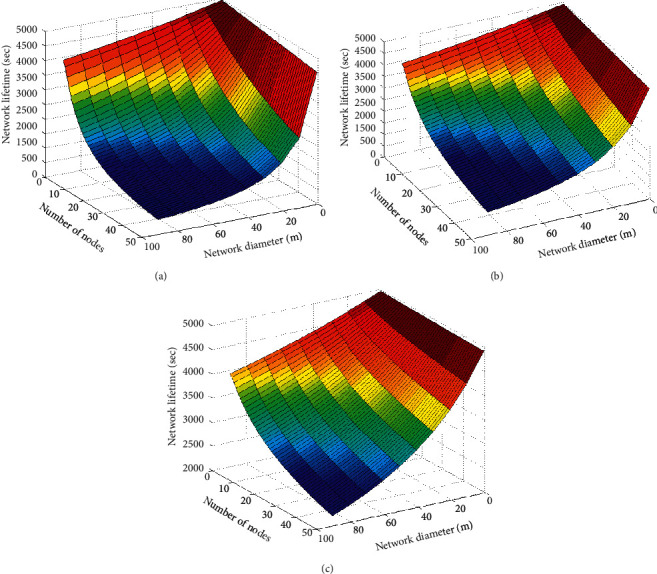
Comparison of the life cycles of the three algorithms. (a) PSO. (b) GWO. (c) SA-GWO.

The network life cycle is one of the important indexes of WSNs network performance. The comparison of the three algorithms is shown in Figure 8.

It can be seen from Figure 8 that, in the same sensing area, when the number of sensor nodes is the same, the sensor node life cycles of PSO algorithm are between 500 s and 4500 s, but half of the sensor node life cycles are below 2500 s, and the overall network life cycle is lower. The sensor node life cycles of the GWO algorithm are between 500 s and 5000 s, but 30% of the sensor node life cycles are below 2500 s, and the overall network life cycle is higher. The sensor node life cycles of SA-GWO algorithm proposed in this paper is between 2000 s and 5000 s, only 20% of sensor node life cycles are below 2500 s, 50% of sensor node life cycles are between 3500 s and 5000 s, and the overall network life cycle is the highest.

#### 5.2.5. The Effect of Communication Radius on Coverage

**Figure 9:**
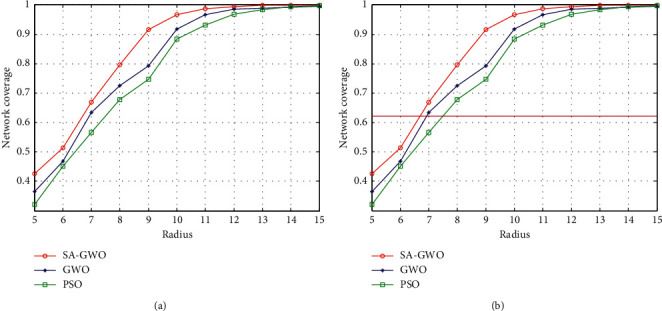
Comparison of the communication radius on coverage of three algorithms.

In order to show the superiority of the algorithm proposed in this paper, we add the experiment of the influence of the communication radius on the network coverage. The comparison results of the algorithms are shown in Figure 9.

As can be seen from Figure 9, when the communication radius of the sensor nodes increases from 5 m to 15 m, the network coverage rate of the three algorithms is gradually increasing, the performance of the proposed SA-GWO algorithm is the best, the GWO algorithm is better, and the performance of the PSO algorithm is poor. It can also be seen that when the communication radius increases from 6 m to 10 m, the network coverage of the three algorithms increases most. Taking the communication radius 9 m as an example, the network coverage rate of PSO algorithm reaches 0.75, the network coverage rate of the GWO algorithm reaches 0.79, and the network coverage rate of SA-GWO algorithm reaches 0.91. It can be seen that the coverage performance of the proposed algorithm is the best.

From the above simulation results, since the SA-GWO algorithm introduces the simulated annealing algorithm, the simulated annealing algorithm is embedded into the wolf pack after the siege behavior ends and before the grey wolf is updated, enhancing the global optimization ability of the grey wolf algorithm, and improves the convergence speed of the grey wolf algorithm. It shows excellent performance in optimizing the coverage of wireless sensor nodes, has superior global optimization ability, and avoids falling into a local optimum. From the above experimental results, we can see that the SA-GWO algorithm proposed in this paper has strong adaptability and fast optimization speed. Applying this algorithm to the coverage optimization of wireless sensor network can significantly improve the network coverage and reduce the coverage blind spots.

## 6. Conclusions

As an important research field of wireless sensor network, in order to solve the coverage problem of wireless sensor network, the probability perception model is used to calculate the network coverage, and a coverage deployment method for wireless sensor network based on grey wolf algorithm optimized by simulated annealing is proposed. The SA-GWO algorithm proposed in this paper can avoid falling into the local optimum, strengthen the global search ability, accelerate the convergence speed of the algorithm, improve the search ability of the algorithm, and improve the coverage rate of the network. To a certain extent, the algorithm proposed in this paper avoids the problem of falling into the local optimum due to the “precocity” of the algorithm in the iterative process and greatly enhances the effectiveness of the deployment of wireless sensor networks.

The SA-GWO algorithm designed in this paper improves the network coverage performance of WSNs to a certain extent, but this paper focuses on the static wireless sensor network. In the later period, it will mainly study the coverage optimization of mobile wireless sensor networks and heterogeneous wireless sensor networks. At the same time, there will be excessive aggregation of nodes in some regions during the application process. The future research direction should make the coverage of WSNs more uniform and reduce the area of node aggregation.

## Data Availability

The data used to support the findings of this study are available from the corresponding author upon request.

## References

[1] Darwish A., Hassanien A. E., Elhoseny M., Sangaiah A. K., Muhammad K. (2019). The impact of the hybrid platform of internet of things and cloud computing on healthcare systems: opportunities, challenges, and open problems. *Journal of Ambient Intelligence and Humanized Computing*.

[2] Yue Y.-G., He P. (2018). A comprehensive survey on the reliability of mobile wireless sensor networks: taxonomy, challenges, and future directions. *Information Fusion*.

[3] Deng X., Jiang Y., Yang L. T., Lin M., Yi L., Wang M. (2019). Data fusion based coverage optimization in heterogeneous sensor networks: a survey. *Information Fusion*.

[4] Sisinni E., Saifullah A., Han S., Jennehag U., Gidlund M. (2018). Industrial internet of things: challenges, opportunities, and directions. *IEEE Transactions on Industrial Informatics*.

[5] Miao Y., Sun Z., Wang N., Cao Y., Cruickshank H. (2018). Time efficient data collection with mobile sink and vMIMO technique in wireless sensor networks. *IEEE Systems Journal*.

[6] Cao L., Cai Y., Yue Y. (2019). Swarm intelligence-based performance optimization for mobile wireless sensor networks: survey, challenges, and future directions. *IEEE Access*.

[7] More A., Raisinghani V. (2017). A survey on energy efficient coverage protocols in wireless sensor networks. *Journal of King Saud University-Computer and Information Sciences*.

[8] Elhabyan R., Shi W., St-Hilaire M. (2019). Coverage protocols for wireless sensor networks: review and future directions. *Journal of Communications and Networks*.

[9] Idrees A. K., Deschinkel K., Salomon M., Couturier R. (2016). Perimeter-based coverage optimization to improve lifetime in wireless sensor networks. *Engineering Optimization*.

[10] Rout M., Roy R. (2016). Self-deployment of mobile sensors to achieve target coverage in the presence of obstacles. *IEEE Sensors Journal*.

[11] Singh R., Manu M. S. (2017). An energy efficient grid based static node deployment strategy for wireless sensor networks. *International Journal of Electronics and Information Engineering*.

[12] Sharma G., Kumar A. (2017). Dynamic range normal bisector localization algorithm for wireless sensor networks. *Wireless Personal Communications*.

[13] Gao Y., Wang J., Wu W., Sangaiah A. K., Lim S.-J. (2019). Travel route planning with optimal coverage in difficult wireless sensor network environment. *Sensors*.

[14] Cao L., Yue Y., Cai Y., Zhang Y. (2021). A novel coverage optimization strategy for heterogeneous wireless sensor networks based on connectivity and reliability. *IEEE Access*.

[15] Cao L., Yue Y., Zhang Y., Cai Y. (2021). Improved crow search algorithm optimized extreme learning machine based on classification algorithm and application. *IEEE Access*.

[16] Yang M., Wang A., Sun G., Zhang Y. (2018). Deploying charging nodes in wireless rechargeable sensor networks based on improved firefly algorithm. *Computers & Electrical Engineering*.

[17] Wang J., Ju C., Gao Y. (2018). A PSO based energy efficient coverage control algorithm for wireless sensor networks. *CMC-computers Materials & Continua*.

[18] Tian J., Gao M., Ge G. (2016). Wireless sensor network node optimal coverage based on improved genetic algorithm and binary ant colony algorithm. *EURASIP Journal on Wireless Communications and Networking*.

[19] Feng Y., Zhao S., Liu H. (2020). Analysis of network coverage optimization based on feedback *K*-means clustering and artificial fish swarm algorithm. *IEEE Access*.

[20] Hashim H. A., Ayinde B. O., Abido M. A. (2016). Optimal placement of relay nodes in wireless sensor network using artificial bee colony algorithm. *Journal of Network and Computer Applications*.

[21] Postorino M. N., Versaci M. (2014). A geometric fuzzy-based approach for airport clustering. *Advances in Fuzzy Systems*.

[22] Cacciola M., Calcagno S., Morabito F. C., Versaci M. (2007). Swarm optimization for imaging of corrosion by impedance measurements in eddy current test. *IEEE Transactions on Magnetics*.

[23] Wang L., Wu W., Qi J., Jia Z. (2018). Wireless sensor network coverage optimization based on whale group algorithm. *Computer Science and Information Systems*.

[24] Li Q., Liu N. (2020). Monitoring area coverage optimization algorithm based on nodes perceptual mathematical model in wireless sensor networks. *Computer Communications*.

[25] Khalaf O. I., Abdulsahib G. M., Sabbar B. M. (2020). Optimization of wireless sensor network coverage using the bee algorithm. *Journal of Information Science and Engineering*.

[26] Mirjalili S., Mirjalili S. M., Lewis A. (2014). Grey wolf optimizer. *Advances in Engineering Software*.

[27] Mirjalili S., Saremi S., Mirjalili S. M., Coelho L. d. S. (2016). Multi-objective grey wolf optimizer: a novel algorithm for multi-criterion optimization. *Expert Systems with Applications*.

[28] Sahoo A., Chandra S. (2017). Multi-objective grey wolf optimizer for improved cervix lesion classification. *Applied Soft Computing*.

[29] Abdel-Basset M., El-Shahat D., El-henawy I., de Albuquerque V. H. C., Mirjalili S. (2020). A new fusion of grey wolf optimizer algorithm with a two-phase mutation for feature selection. *Expert Systems with Applications*.

[30] Li X., Luk K. M. (2019). The grey wolf optimizer and its applications in electromagnetics. *IEEE Transactions on Antennas and Propagation*.

[31] Jiao Z., Zhang J., Yao P., Wan L., Ni L. (2020). Service deployment of C4ISR based on genetic simulated annealing algorithm. *IEEE Access*.

[32] Duan Z., Wei X., Han J., Lu Y., Shi L. (2020). Simulated annealing-based reprogramming scheme of wireless sensor nodes. *Wireless Networks*.

